# Outcomes of Critically Ill Adult Patients With Acute Encephalitis

**DOI:** 10.1001/jamanetworkopen.2025.32478

**Published:** 2025-09-18

**Authors:** Romain Sonneville, Camille Couffignal, Bertrand Souweine, Suela Demiri, Nicolas Terzi, Fabrice Bruneel, Armand Mekontso Dessap, Maelle Martin, Clémence Marois, Pierre Bailly, Achille Kouatchet, Guillaume Voiriot, Florian Reizine, Sarah Benghanem, Guillaume Louis, Marie Conrad, Sami Hraiech, Arnaud W. Thille, Jean-Christophe Navellou, Damien Contou, Simon Bourcier, Marc Tran, Frederic Dailler, Laurent Argaud, Charles Cerf, Bertrand Guidet, Damien Roux, Lionel Kerhuel, Guillaume Van Der Meersch, Saad Nseir, Adrien Joseph, Daniel Da Silva, Pascal Beuret, Benjamine Sarton, Candice Estellat, Virginie Godard, Jérôme Honnorat, Philippa Catherine Lavallée, Marina Esposito-Farèse, Jean-François Timsit

**Affiliations:** 1 Université Paris Cité, IAME, INSERM UMR1137, Paris, France; 2 Service de Médecine Intensive-Réanimation, Assistance Publique-Hôpitaux de Paris, Hôpital Bichat-Claude Bernard, Paris, France; 3 Unité de Recherche Clinique, Assistance Publique-Hôpitaux de Paris, Hôpital Bichat-Claude Bernard, Paris, France; 4 Médecine Intensive Réanimation, Centre Hospitalier Universitaire Gabriel Montpied, Clermont Ferrand, France; 5 Service de Médecine Intensive et Réanimation Département R3S, Sorbonne Université, INSERM, UMRS1158, Neurophysiologie Respiratoire Expérimentale et Clinique, Assistance Publique-Hôpitaux de Paris, Groupe Hospitalier Universitaire Assistance Publique-Hôpitaux de Paris-Sorbonne Université, site Pitié-Salpêtrière, Paris, France; 6 Médecine Intensive-Réanimation, Grenoble Alpes Université, INSERM 1300, HP2, Centre Hospitalier Universitaire Grenoble Alpes, Grenoble, France; 7 Médecine Intensive Réanimation, Centre Hospitalier de Versailles, Hôpital André Mignot, Le Chesnay-Rocquencourt, France; 8 Médecine Intensive Réanimation, Assistance Publique-Hôpitaux de Paris, Centre Hospitalier Universitaire Henri Mondor, Créteil, France; 9 Médecine Intensive Réanimation, Centre Hospitalier Universitaire de Nantes, Nantes, France; 10 Médecine Intensive Réanimation à Orientation Neurologique, Sorbonne Université Assistance Publique-Hôpitaux de Paris, Hôpital Pitié-Salpêtrière, Paris, France; 11 Médecine Intensive Réanimation, Centre Hospitalier Universitaire de la Cavale Blanche, Brest, France; 12 Médecine Intensive Réanimation, Centre Hospitalier Universitaire d’Angers, Angers, France; 13 Service de Médecine Intensive Réanimation, Sorbonne Université, Assistance Publique-Hôpitaux de Paris, Hôpital Tenon, Centre de Recherche Saint-Antoine UMRS_938 INSERM, Paris, France; 14 Médecine Intensive Réanimation, Centre Hospitalier de Vannes, Vannes, France; 15 Médecine Intensive Réanimation, Université Paris Cité, Assistance Publique-Hôpitaux de Paris Centre, Hôpital Cochin, Paris, France; 16 Réanimation Polyvalente, Centre Hospitalier Régional Metz-Thionville, Hôpital de Mercy, Metz, France; 17 Médecine Intensive Réanimation, Centre Hospitalier Universitaire de Nantes, Nantes, France; 18 Médecine Intensive Réanimation, Assistance Publique-Hôpitaux de Marseille, Marseille, France; 19 Médecine Intensive Réanimation, Centre Hospitalier Universitaire de Poitiers, Poitiers, France; 20 Service de Réanimation Médicale, Centre Hospitalier Universitaire de Besançon, Besançon, France; 21 Réanimation Polyvalente, Centre Hospitalier Victor Dupouy, Argenteuil, France; 22 Médecine Intensive Réanimation, Assistance Publique-Hôpitaux de Paris, Hôpital Pitié–Salpêtrière, Paris, France; 23 Réanimation, Groupe Hospitalier Paris Saint-Joseph, Paris, France; 24 Réanimation Neurologique, Groupement Hospitalier Est, Hospices Civils de Lyon, Lyon, France; 25 Médecine Intensive-Réanimation, Hospices Civils de Lyon, Hôpital Edouard Herriot, Lyon, France; 26 Réanimation Polyvalente, Hôpital Foch, Suresnes, France; 27 Sorbonne Université, INSERM, Institut Pierre Louis d’Épidémiologie et de Santé Publique, Équipe PEPITES, Assistance Publique-Hôpitaux de Paris, Hôpital Saint Antoine, Paris, France; 28 Médecine Intensive Réanimation, Assistance Publique-Hôpitaux de Paris, Hôpital Louis Mourier, DMU ESPRIT, Colombes, Université Paris Cité, INSERM, Centre National de la Recherche Scientifique, Institut Necker-Enfants Malades, Paris, France; 29 Médecine Intensive Réanimation, Assistance Publique-Hôpitaux de Paris, Hôpital Saint Louis, Paris, France; 30 Médecine Intensive Réanimation, Assistance Publique-Hôpitaux de Paris, Hôpital Avicenne, Bobigny, France; 31 Médecine Intensive Réanimation, Hôpital Roger Salengro, Centre Hospitalier Universitaire de Lille, Lille, France; 32 Médecine Intensive Réanimation, Assistance Publique-Hôpitaux de Paris, Hôpital Ambroise Paré, Boulogne, France; 33 Médecine Intensive Réanimation, Centre Hospitalier Delafontaine, Saint Denis, France; 34 Service de Réanimation et Soins Continus, Centre Hospitalier de Roanne, Roanne, France; 35 Réanimation Polyvalente, Centre Hospitalier Universitaire de Toulouse, Hôpital Purpan, Toulouse, France; 36 Unité de Recherche Clinique, Assistance Publique-Hôpitaux de Paris, Université Paris Sorbonne, GH Pitié Salpêtrière, Paris, France; 37 Centre de Référence des Syndromes Paranéoplasiques et Encéphalites Autoimmunes, Hospices Civils de Lyon, MeLiS-UCBL-CNRS UMR 5284, INSERM U1314, Université Claude Bernard Lyon 1, Lyon, France; 38 Service de Neurologie, Université Paris Cité, Assistance Publique-Hôpitaux de Paris, Hôpital Bichat-Claude Bernard, Paris, France

## Abstract

**Importance:**

Functional outcomes and long-term recovery after severe encephalitis are not well characterized.

**Objective:**

To determine the incidence of functional disability or death at 3 months and to describe recovery trajectories through 1 year after encephalitis.

**Design, Setting, and Participants:**

This prospective multicenter cohort study was conducted across 31 French centers from October 2017 to April 2021 and included adults with probable or confirmed encephalitis and clear cerebrospinal fluid findings requiring care in the intensive care unit. Data analysis was performed between May 2023 and June 2025.

**Exposure:**

Causes of encephalitis were categorized into 4 different groups: infectious, autoimmune, other causes, and unknown origin.

**Main Outcomes and Measures:**

The primary end point was an unfavorable outcome at 3 months, defined by a modified Rankin scale score of 3 to 6, indicating moderate to severe disability or death.

**Results:**

Among the 310 patients included (median [IQR] age, 60 [43-70] years; 177 male [57.1%]), 123 (39.7%) were diagnosed with infectious encephalitis, 42 (13.5%) with autoimmune encephalitis, 37 (11.9%) with other encephalitis causes, and 108 (34.8%) with encephalitis of unknown origin. Overall, 161 patients (51.9%; 95% CI, 46.2%-57.6%) had an unfavorable outcome at 3 months, including 84 deaths (27.1%). Independent factors associated with unfavorable outcome included age (odds ratio [OR] per 5-year increment, 1.28, 95% CI, 1.16 to 1.41) and immunocompromised status (OR, 3.12; 95% CI, 1.57 to 6.40), while intravenous acyclovir on the day of ICU admission was associated with a favorable outcome (OR, 0.38; 95% CI, 0.20 to 0.72). The proportion of patients achieving functional independence remained stable from 3 months to 1 year (difference in proportions, 1.1%; 95% CI, −6.9% to 9.2%). Analyses based on encephalitis cause groups revealed that patients with autoimmune encephalitis showed significant improvement through 1 year (difference in proportions, 8.9%; 95% CI, 1.2% to 16.6%), whereas no significant changes were seen in patients with infectious causes (difference in proportions, 1.2%; 95% CI, −6.9% to 9.2%), other causes (difference in proportions, 1.2%; 95% CI: −6.8% to 9.2%), or unknown origin (difference in proportions, −1.9%; 95% CI: −10.0% to 6.2%).

**Conclusions and Relevance:**

In this cohort study of adults with severe encephalitis requiring intensive care, one-half of patients had an unfavorable outcome at 3 months. Functional recovery at 1 year varied by cause of encephalitis, with patients with autoimmune encephalitis experiencing more favorable outcomes than those with other causes, suggesting a possible role for targeted long-term support in certain cases.

## Key Points

**Question:**

What are the long-term functional outcomes of adults with severe encephalitis, and how do recovery trajectories differ by cause of encephalitis?

**Findings:**

In this cohort study of 310 adults with probable or confirmed encephalitis, all with clear cerebrospinal fluid findings and requiring intensive care, 51.9% had an unfavorable outcome at 3 months, including mortality among 27.1% of patients. Functional outcomes remained largely unchanged at 1 year, except among patients with autoimmune encephalitis, who demonstrated improvement.

**Meaning:**

In this cohort study, one-half of adult patients with severe encephalitis had a poor prognosis at 3 months, with significant variability in recovery trajectories at 1 year depending on the cause of encephalitis, suggesting a possible role for targeted long-term support in certain cases.

## Teaser

This cohort study describes the incidence of moderate to severe disability or death at 3 months among adults with encephalitis admitted to intensive care and explores recovery trajectories among these patients up to 1 year.

## Introduction

Encephalitis is an inflammatory brain condition commonly caused by infectious pathogens and, less frequently, by autoimmune disorders, toxins, or other diseases.^1^ Patients with encephalitis develop an acute onset encephalopathy with a combination of fever, focal signs and/or new-onset seizures, together with cerebrospinal fluid (CSF) pleocytosis, brain imaging features of inflammation, and/or electroencephalography (EEG) alterations.^2^

Herpes simplex encephalitis (HSE) remains the most common cause of sporadic infectious encephalitis in immunocompetent adults, and is associated with a severe prognosis.^3,4,5,6,7^ The causal landscape of encephalitis has shifted substantially, with increasing rates of autoimmune causes such as acute disseminated encephalomyelitis (ADEM) and anti-N-methyl-D-aspartate receptor (anti-NMDAR) encephalitis, which often necessitate intensive care and urgent immunomodulatory treatment.^8,9,10^ New diagnostic methods such as multiplex polymerase chain reaction and metagenomics have improved the diagnosis of uncommon infectious causes of encephalitis,^11,12^ but cases of unknown origin remain as high as 30% to 48%.^13,14,15^

Epidemiological studies indicate that approximately 40% of adult encephalitis cases require intensive care unit (ICU) admission.^6,16^ In a French multicenter study, ICU admission was independently associated with poorer functional outcomes.^7^ Single-center studies suggest that patients with encephalitis who require ICU admission have a severe prognosis in the short term, including a higher risk of refractory seizures, prolonged hospitalization, disability, and death.^13,14,15,17^ Recently, a large prospective international multicenter cohort study conducted in patients with all-cause adult meningoencephalitis requiring ICU admission reported a severe short term prognosis in one-half of cases, with a 3-month mortality rate of 25%.^18^ In the present study, we aimed to characterize functional outcomes and recovery trajectories through 1 year among ICU-admitted adults with probable or confirmed encephalitis and clear CSF findings.

## Methods

### Study Design and Population

The Encephalitis in Intensive Care (ENCEPHALITICA) study is a prospective multicenter cohort study registered in ClinicalTrials.gov (NCT02906631) and conducted across 31 French ICUs from October 26, 2017, to April 7, 2021. Ethical approval was obtained from the Comité de Protection des Personnes Est 3 (May 8, 2017). The study is reported according to the Strengthening the Reporting of Observational Studies in Epidemiology (STROBE) reporting guideline.^19^ Eligible patients were adults with suspected or confirmed encephalitis according to the Infectious Diseases Society of America 2013 criteria.^2^ Inclusion criteria were: (1) adult patients (≥18years); (2) admission to the ICU for acute encephalopathy (ie, altered mental status, delirium, or personality changes for a duration ≥24 hours); (3) a score of 13 or less on the Glasgow Coma Scale (GCS) in the absence of sedation; (4) a CSF analysis performed; (5) brain imaging performed; and (6) at least 2 of the following criteria: (1) fever (≥38 °C) within 72 hours before or following admission; (2) new-onset seizures; (3) new-onset focal signs; (4) CSF pleocytosis (≥5 cell/µL); (5) brain parenchyma abnormalities on neuroimaging compatible with the diagnosis of encephalitis; and (6) EEG abnormalities compatible with the diagnosis of encephalitis. Exclusion criteria were (1) time between first neurologic symptoms and ICU admission greater than 21 days; (2) community a bacterial meningitis (ie, purulent CSF or positive CSF Gram staining or culture) due to *Streptococcus pneumoniae* or *Neisseria meningitidis*; (3) brain abscess on neuroimaging; (4) febrile encephalopathy explained by another likely diagnosis; and (4) an expected length of stay in ICU (24 hours or less). Written informed consent was obtained from the patient or next of kin at inclusion. For patients unable to consent at inclusion who eventually recovered, a written informed consent was obtained during follow-up. For the present study, we secondarily excluded patients with missing baseline data, missing modified Rankin scale (mRS) score at 3 months, and those who withdrew consent during follow-up.

### Adjudication Committee

An independent adjudication committee composed of 3 senior neurologists, each certified in intensive care medicine, conducted a review of all included cases. Two investigators examined the medical records of the patients to assess clinical, imaging, and EEG diagnostic criteria for encephalitis, causes of encephalitis, as well as the evaluation of the primary end point at 3 months. In instances where discrepancies arose between the 2 investigators, a third investigator was consulted to review the conflicting cases. Causes of encephalitis were categorized into 4 different groups: infectious, autoimmune, other causes, and unknown origin. A definition of each groups is provided in eTable 1 in Supplement 1.

### Data Collection

Epidemiological data was prospectively recorded in a secure, web-based case record form. Demographics, premorbid mRS score, chronic immunodepression (ie, HIV infection, solid organ transplantation recipient, patient with cancer or hematological condition, and long-term use of steroids and/or immunosuppressant drugs), preexisting neurologic disease, and other comorbidities were collected, together with ICU admission data. Data collected within the first 24 hours following ICU admission included the main reason for ICU admission, the Simplified Acute Physiology Score 2 (SAPS2),^20^ and detailed components of the GCS^21^ and the sequential organ failure assessment (SOFA) scores.^22^ Coma was defined as a GCS score less than 8,^23^ and fever was defined as a body temperature of 38 °C or greater.^2^ Acute respiratory failure and cardiovascular failure were defined as a respiratory and cardiovascular component of the SOFA score greater than 2.^22^ For each patient, biological samples (serum and CSF) were collected at inclusion and at 14 days, and stored at −80 °C. Imaging files (Digital Imaging and Communications in Medicine) and EEG files (European data format) were prospectively collected and stored by coordinating investigators for ancillary studies. For each included patient, we collected daily ICU clinical data from inclusion to day 7 and clinical status at day 14, day 28, day 60, day 90, and at 1 year.

### Treatment

Standardized investigations (ie, CSF and serum analyses, imaging, EEG, oriented samples in case of non-neurological manifestations) were recommended to investigators for patients without clear causal diagnoses, following the Infectious Diseases Society of America encephalitis guidelines.^2^ Additional investigations were suggested in other circumstances, such as immunocompromised status, specific clinical presentation (ie, psychiatric signs or abnormal movements), or, depending on topography of brain lesions, on neuroimaging. Patients were treated empirically and, depending on the identified cause, according to local guidelines. Dose and duration of antimicrobials (ie, intravenous acyclovir and antibiotics) and/or immunomodulating agents (ie, intravenous steroids, intravenous immunoglobulins, and plasma exchange), and prescriptions of antiseizure drugs were left to the discretion of investigators.

### End Points

The primary end point was an unfavorable outcome at 3 months postinclusion, defined by an mRS score of 3 to 6, indicating moderate to severe disability or death. Causes of death were categorized into 2 groups: systemic causes (such as cardiovascular failure or multiple organ failure) and neurological causes (such as withdrawal of care due to diffuse neurological injury or brain death). Secondary end points included the proportion of patients achieving functional independence at specific time points (ICU discharge, 3 months, and 1 year postinclusion), as well as the place of residence at specific time points (day 14, day 28, day 60, day 90, and 1 year postinclusion). Functional independence was defined by a score on the mRS of 0 to 2. Places of residence in survivors were classified into 4 categories: home, follow-up care or rehabilitation, medical wards, and ICU. All end points were prospectively collected via telephone interviews by research assistants trained for scoring on the mRS.^24,25^ When patients were unable to be evaluated directly, evaluations were performed with help of family members or professional caregivers, as appropriate.

### Statistical Analysis

Data were analyzed from May 2023 to June 2025. Continuous variables are presented as medians with IQRs, and categorical variables as frequencies and percentages. Comparisons of continuous variables were performed using the Wilcoxon rank sum test with normal approximation and continuity correction. Categorical variables were compared using the χ^2^ test or Fisher exact test, as appropriate. Differences in proportions between 3-month and 1-year outcomes were calculated, and 95% CIs were derived using the Miettinen and Nurminen method.

For multivariable logistic regression, baseline variables associated with the unfavorable outcome were selected based on clinical relevance and prior literature. Two models were constructed: one including all selected variables, and a second using stepwise selection. Collinearity among variables was assessed prior to model development. Variables with *P* < .10 in univariable analyses were considered for inclusion. All tests were 2-sided, with *P* < .05 considered statistically significant. Analyses were performed using R version 4.4 (R Foundation for Statistical Computing).

## Results

### Study Population

From October 26, 2017, to April 7, 2021, we identified 352 eligible patients from 31 centers, with a median (IQR) of 8 (3-14) patients per center (eTable 2 in Supplement 1). Among them, 4 patients were excluded by the adjudication committee and 348 patients were registered in the ENCEPHALITICA cohort. A total of 310 patients with an available mRS score at 3 months were included in the present study (eFigure in Supplement 1). A description of the 38 excluded patients is provided in eTable 3 in Supplement 1. Characteristics of patients at inclusion are presented in Table 1. The median (IQR) age was 60 (43-70) years, and 177 (57.1%) were male. Patients had frequent comorbidities, with a median (IQR) Charlson Comorbidity Index score of 3 (1-6), and 74 (23.9%) had an immunocompromised status. The median (IQR) time between onset of neurologic symptoms and hospitalization was 1 (0-2) days, and the median (IQR) time from hospital admission to ICU transfer was 0 (0-2) days. At ICU admission, patients had severe encephalopathy with a median (IQR) score on the GCS of 10 (7-12), and 154 of 309 patients (49.8%) were intubated. Other key clinical findings included a combination of convulsive seizures (142 patients [45.8%]), focal neurologic findings (163 patients [52.6%]), and fever (293 patients [77.1%]). Patients were severely ill, as reflected by a median (IQR) SAPS2 score of 42 (29-45) and a median (IQR) non-neurological SOFA score of 3 (1-5). CSF analysis and neuroimaging were performed in all included patients early during encephalitis. Neuroimaging included a computed tomography scan in 266 of 309 cases (86.1%), MRI in 223 of 309 cases (72.2%) cases, and a combination of both in 182 of 309 cases (58.9%). Finally, EEG was performed in 273 of 308 patients (88.6%). Compared with other patients, patients with autoimmune encephalitis were younger, had fewer comorbidities, fewer frequent seizure events, and less frequent non-neurological failure at inclusion.

**Table 1.** Patient Characteristics at Inclusion

Characteristic	Participants by encephalitis cause, No./total No. (%)					*P* value
	Total (N = 310)	Infectious (n = 123)	Autoimmune (n = 42)	Other (n = 37)	Unknown (n = 108)	
Age, median (IQR), y	60 (43 to 72)	58 (44 to 71)	42 (26 to 58)	69 (56 to 77)	65 (48 to 75)	<.001
Sex						
Male	177/310 (57.1)	79/123 (64.2)	21/42 (50)	19/37 (51.4)	58/108 (53.7)	.22
Female	133/310 (42.9)	44/123 (35.8)	21/42 (50)	18/37 (8.6)	50/108 (46.2)	
Charlson Comorbidity Index score, median (IQR)	3 (1 to 6)	3 (1 to 6)	1 (0 to 4)	4 (3 to 7)	4 (2 to 5)	<.001
Immunodepression^a^	74/310 (23.9)	43/123 (35)	4/42 (9.5)	8/37 (21.6)	19/108 (17.6)	.002
Reason for ICU admission						
Altered mental status	178/309 (57.6)	76/123 (61.8)	21/42 (50)	23/37 (62.2)	58/107 (54.2)	.44
Seizures or status epilepticus	69/309 (22.3)	20/123 (16.3)	12/42 (28.6)	3/37 (8.1)	34/107 (31.8)	.003
Sepsis or respiratory failure	10/309 (3.2)	4/123 (3.3)	0	3/37 (8.1)	3/107 (2.8)	.24
Other	38/309 (12.3)	20/123 (16.3)	7/42 (16.7)	3/37 (8.1)	8/107 (7.5)	.14
Glasgow Coma Scale score, median (IQR)						
Overall score	10 (7 to 12)	10 (7 to 12)	10 (7 to 12)	9 (6 to 11)	9 (6 to 11)	.072
Eye component	3 (1 to 4)	3 (2 to 4)	3 (1 to 4)	3 (2 to 4)	3 (1 to 4)	.72
Verbal component	2 (1 to 3)	2 (1 to 3)	2 (1 to 3)	1 (1 to 2)	2 (1 to 3)	.25
Motor component	5 (4 to 6)	5 (4 to 6)	5 (4 to 6)	4 (3 to 5)	5 (4 to 5)	.008
Temperature						
Median (IQR) °C	37.8 (36.9 to 38.7)	37.9 (37.1 to 38.9)	37 (36.8 to 38.2)	37.5 (36.5 to 38.7)	37.8 (36.8 to 38.6)	.11
Fever (≥38.0 °C)	239/310 (77.1)	102/123 (82.9)	27/42 (64.3)	22/37 (59.5)	88/108 (81.5)	.004
Seizures	142/310 (45.8)	50/123 (40.7)	23/42 (54.8)	14/37 (37.8)	55/108 (50.9)	.19
Focal neurologic findings	163/310 (52.6)	60/123 (48.8)	25/42 (59.5)	22/37 (59.5)	56/108 (51.9)	.53
SAPS 2 score, median (IQR)	42 (29 to 57)	42 (29 to 58)	34 (24 to 44)	56 (38 to 65)	43 (32 to 57)	<.001
Non-neurologic SOFA score, median (IQR)	3 (1 to 5)	3 (1 to 5)	1 (0 to 3)	3 (2 to 7)	3 (1 to 5)	<.001
CSF						
Time from inclusion to lumbar puncture, median (IQR), d	0 (0 to 1)	0 (0 to 1)	0 (0 to 1)	1 (0 to 1)	0 (0 to 1)	.013
CSF leucocytes ≥5 cells/mm^3^	221/277 (79.8)	91/110 (82.7)	31/38 (81.6)	24/32 (75)	75/97 (77.3)	.66
Leucocytes, median IQR, cells/mm^3^	18 (6 to 93)	53 (9 to 165)	14.5 (7 to 37)	10 (5 to 34)	14 (5 to 95)	.003
Lymphocytes, median (IQR), %	74 (17 to 92)	72 (26 to 94)	87 (75 to 96)	72 (10 to 96)	66 (7 to 90)	.11
Protein level, median (IQR), g/dL	0.07 (0.05 to 0.13)	0.09 (0.06 to 0.18)	0.06 (0.04 to 0.09)	0.05 (0.04 to 0.06)	0.07 (0.05 to 0.11)	<.001
Glucose, median (IQR), mg/dL	70.27 (59.46 to 90.09)	66.67 (50.45 to 84.68)	70.27 (59.46 to 86.49)	77.48 (64.86 to 91.89)	75.68 (61.26 to 106.31)	.02
Brain imaging						
CT scan performed	266/309 (86.1)	111/123 (90.2)	34/42 (81)	32/37 (86.5)	89/107 (83.2)	.30
Time from inclusion to CT scan, median (IQR), d	0 (−1 to 0)	0 (−1 to 0)	0 (−1 to 0)	0 (−0.2 to 0)	0 (0 to 0)	.18
Abnormal CT scan	85/263 (32.3)	48/110 (43.6)	5/34 (14.7)	8/31 (25.8)	24/88 (27.3)	.005
MRI performed	223/309 (72.2)	87/123 (70.7)	36/42 (85.7)	25/37 (67.6)	75/107 (70.1)	.18
Time from inclusion to MRI, median (IQR), d	1 (0 to 2)	0 (0 to 2)	0 (−1 to 2)	2 (1 to 3)	1 (0 to 3)	.006
Abnormal MRI	162/223 (72.6)	65/87 (74.7)	26/36 (72.2)	18/25 (72)	53/75 (70.7)	.95
EEG						
EEG performed	273/308 (88.6)	101/123 (82.1)	39/42 (93.0)	33/37 (89.2)	100/108 (92.6)	.03
Time from inclusion to EEG, median (IQR), d	1 (0 to 1)	0 (0 to 1)	1 (0 to 2)	1 (0 to 2)	0.5 (0 to 1)	.15
Abnormal EEG	142/310 (45.8)	52/123 (42.3)	26/42 (61.9)	14/37 (37.8)	50/108 (46.3)	.12

Abbreviations: CSF, cerebrospinal fluid; CT, computed tomography; EEG, electroencephalography; ICU, intensive care unit; MRI, magnetic resonance imaging; SAPS, Simplified Acute Physiology score; SOFA, sequential organ failure assessment.

SI conversion factors: To convert glucose to millimoles per liter, multiply by 0.0555; protein to g/L, multiply by 10.

^a^ Immunodepression includes patients living with HIV (25 individuals), solid organ transplantation (17 individuals), cancer or hematology disease (25 individuals), and/or steroids/immunosuppressants (27 individuals).

### Encephalitis Causes

Causes of encephalitis and their association with outcomes at 3 months are presented in Table 2. Among the 310 included patients, 123 (39.7%) had an infectious cause, including 33 (10.6%) with herpes simplex virus, 15 (4.8%) with varicella-zoster virus, and 10 (3.2%) with mycobacterium tuberculosis. Autoimmune encephalitis was identified in 42 patients (13.5%), while 37 patients (11.9%) had other causes, and 108 (34.8%) had an unknown cause. We observed no significant association of encephalitis causes with outcomes at 3 months.

**Table 2.** Causes of Encephalitis

Encephalitis cause	Participants, No. (%)		
	Total (N = 310)	Immunocompetent (n = 236)	Immunocompromised (n = 74)
Infectious			
Overall	123 (39.6)	80 (33.9)	43 (58.1)
Herpes simplex virus 1	33 (10.6)	26 (11.0)	7 (9.5)
Varicella zoster virus	15 (4.8)	10 (4.2)	5 (6.8)
Mycobacterium tuberculosis	10 (3.2)	9 (3.8)	1 (1.4)
Listeria monocytogenes	4 (1.3)	3 (1.3)	1 (1.4)
Other infectious^a^	61 (19.7)	32 (13.5)	29 (39.2)
Autoimmune			
Overall	42 (13.5)	38 (16.1)	4 (5.4)
Anti-NMDAR	8 (3.2)	8 (3.4)	0
Acute disseminated encephalomyelitis	4 (1.3)	3 (1.3)	1 (1.4)
Other autoimmune^b^	30 (9.7)	27 (11.4)	3 (4.1)
Other causes^c^	37 (11.9)	29 (12.3)	8 (10.8)
Unknown origin	108 (34.8)	89 (37.7)	19 (25.7)

Abbreviation: NMDAR, N-methyl-D-aspartate receptor.

^a^ Toxoplasmosis (6 individuals), HIV (6 individuals), plasmodium falciparum (4 individuals), streptococcus (3 individuals), staphylococcus (3 individuals), herpes simplex virus 2 (4 individuals), Epstein Barr virus (4 individuals), *Escherichia coli* (3 individuals), West Nile virus (2 individuals), syphilis (3 individuals), human herpesvirus 6 (3 individuals), cytomegalovirus (3 individuals), Tropheryma whipplei (1 individual), Proteus mirabilis (1 individual), polymicrobial (1 individual), legionella (1 individual), enterovirus (1 individual), cryptococcus (1 individual), Creutzfeldt-Jakob (1 individual), Cladothialophoria bantiana (1 individual), aspergillus (1 individual), adenovirus (1 individual), actinomyces (1 individual), and other (6 individuals).

^b^ Rheumatic disease (4 individuals), post–COVID-19 condition (4 individuals), paraneoplastic (3 individuals), antiglial fibrillary acidic protein (2 individuals), post mycoplasma pneumoniae (2 individuals), Hashimoto encephalopathy (1 individual), Bickerstaff (n = 1 individual), anti-CV2 and collapsin response-mediator protein 5 (1 individual), anti–leucine-rich glioma-inactivated 1 (1 individual), antiglutamic acid decarboxylase (1 individual), anti–gamma-aminobutyric acid type B receptor (1 individual), and other antibody negative (n = 9 individuals).

^c^ Metabolic or toxic (28 individuals) and neoplastic (9 individuals).

### ICU Treatment

Within 24 hours of ICU admission, 204 patients (65.8%) received intravenous acyclovir, and 106 (34.2%) received third generation cephalosporins. During ICU stay, 71 of 309 patients (22.9%) received adjunctive steroids, 31 of 308 patients (10.1%) received intravenous immunoglobulins, and 14 of 308 patients (4.5%) received plasma exchange.

A description of treatments given during ICU stay is provided in the eTable 4 in Supplement 1. Overall, 227 of 309 patients (73.5%) required invasive mechanical ventilation during ICU stay, for a median (IQR) duration of 6 (6-12) days. The median (IQR) ICU length of stay was 9 (5-16) days, and decisions to withhold or withdraw care during ICU stay were reported in 46 of 308 patients (14.9%) during ICU stay. Discharge outcomes in ICU survivors included medical wards (238 of 256 patients [93.0%]), follow-up care or rehabilitation (15 of 256 patients [5.9%]), and home (3 of 256 patients [1.2%]).

The duration of mechanical ventilation and ICU stay varied substantially by cause of encephalitis. Notably, patients with autoimmune encephalitis experienced prolonged mechanical ventilation and ICU admission compared with other causes. Additionally, these patients were more frequently prescribed acyclovir and received immunotherapies—including corticosteroids, intravenous immunoglobulins, and plasma exchange—more often than other groups. Finally, decisions to withhold or withdraw care during ICU stay were similar across encephalitis causes.

### Study End Points

**Figure 1.**
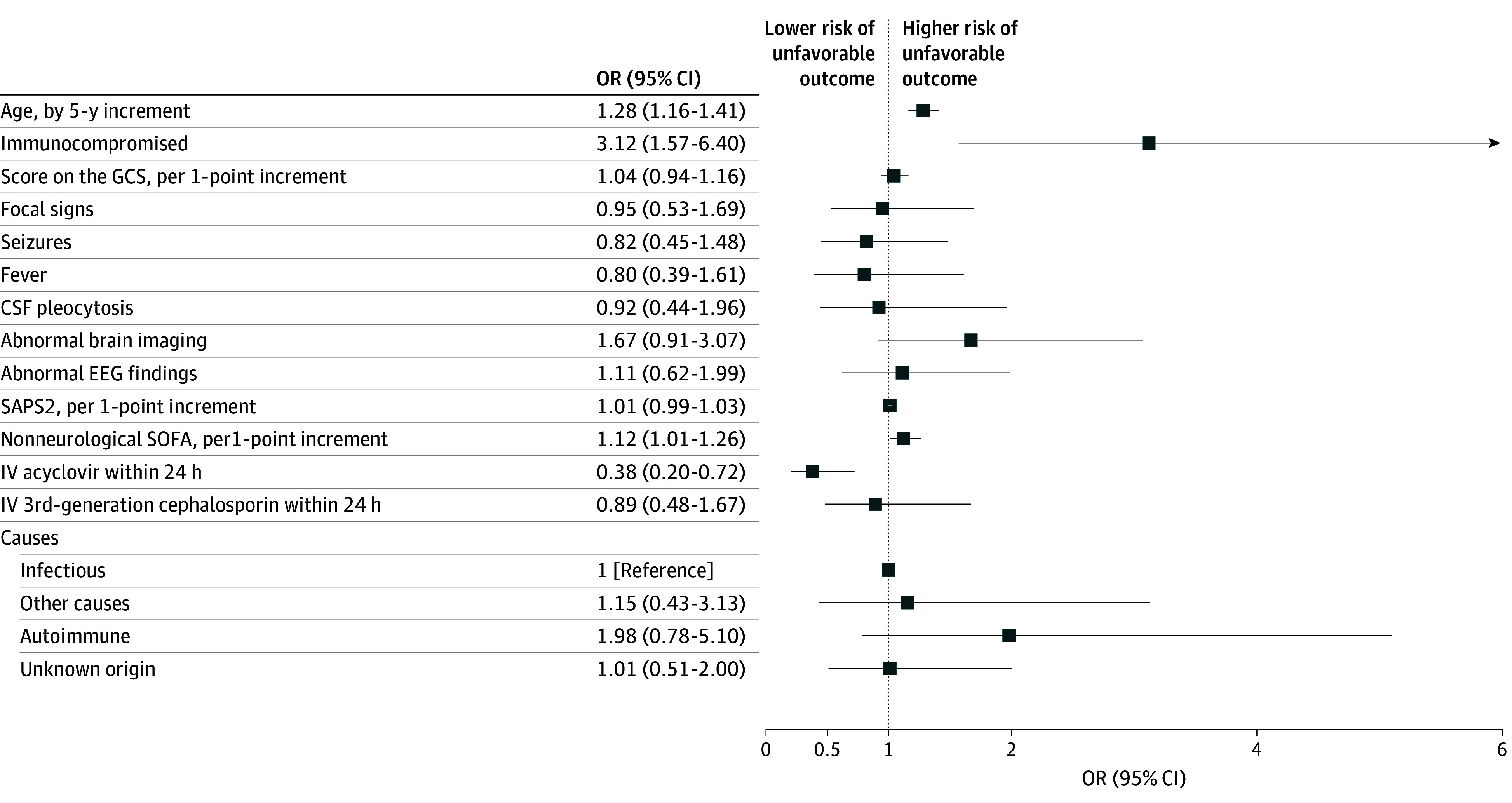
Factors Associated With an Unfavorable Outcome, Multivariable Analysis An unfavorable outcome was moderate to severe disability or death, defined by a modified Rankin score of 3 to 6. CSF indicates cerebrospinal fluid; EEG, electroencephalography; GCS, Glasgow Coma Scale; IV, intravenous; OR, odds ratio; SAPS, Simplified Acute Physiology score; SOFA, sequential organ failure assessment.

At 3 months, 161 patients (51.9%; 95% CI, 46.2%-57.6%) had an unfavorable outcome, including 84 deaths (27.1%). Causes of death included neurologic causes (40 of 84 patients [47.6%]; including 5 brain deaths), systemic causes (27 of 84 patients [32.1%]), and undetermined cause in 12 of 84 cases (14.2%). Baseline characteristics and their association with an unfavorable outcome at 3 months are presented in eTable 5 in Supplement 1. Independent factors associated with an unfavorable outcome in the full model included age per 5-year increment (odds ratio [OR], 1.28; 95% CI, 1.16-1.41) and immunocompromised status (OR, 3.12, 95% CI, 1.57-6.40), while early intravenous acyclovir on the day of ICU admission was associated with a favorable outcome (OR, 0.38; 95% CI, 0.20-0.72) (Figure 1). The multivariable analysis using a stepwise method yielded similar results (eTable 6 in Supplement 1).

**Figure 2.**
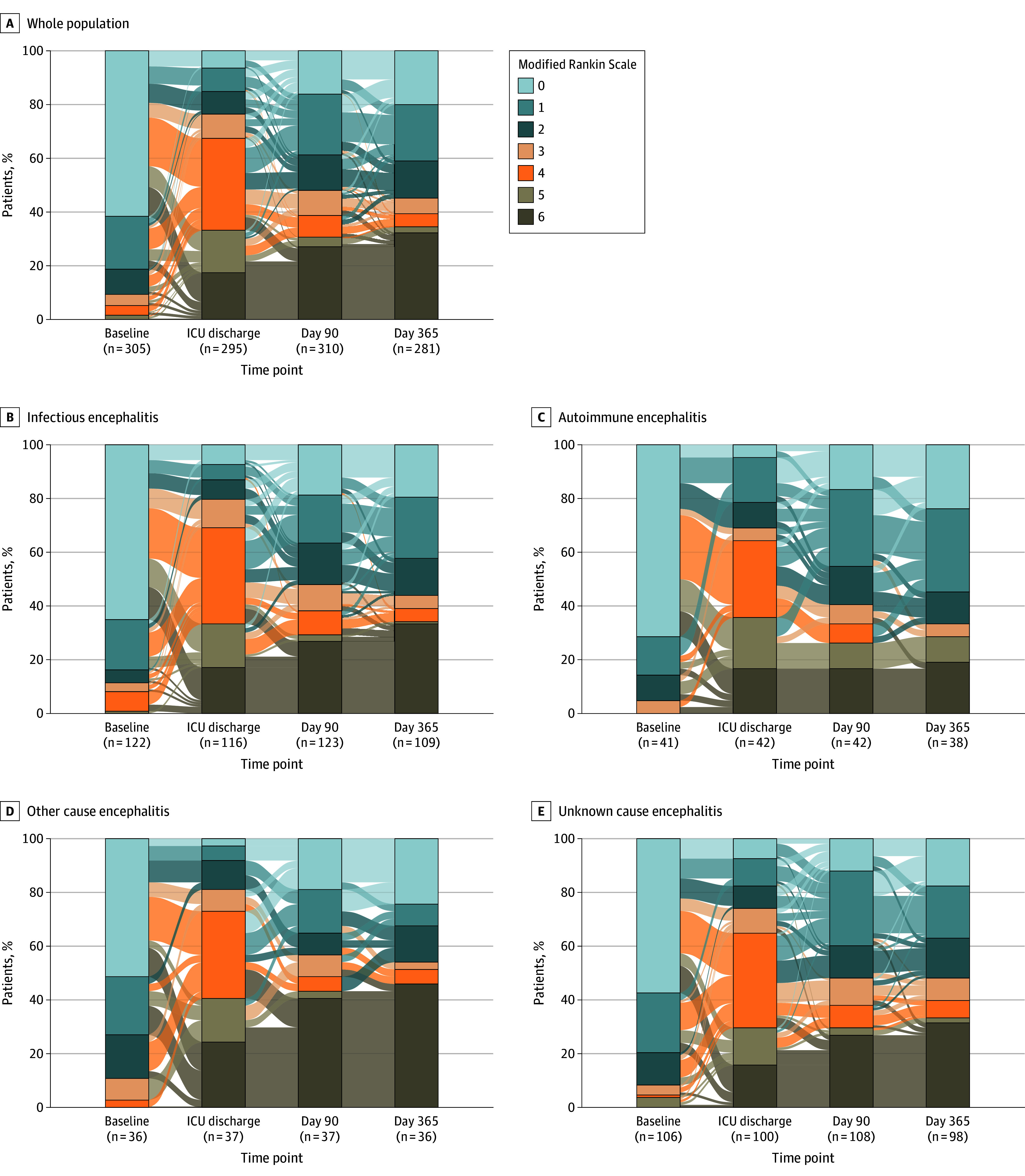
Patient Functional Status on the Modified Rankin Scale Up to 1 Year After Severe Encephalitis The lines between each bar represent changes in Rankin score between time points. ICU indicates intensive care unit.

The distribution of mRS scores at various time points for the entire cohort, as well as for the different encephalitis cause groups (infectious, autoimmune, other causes, and unknown), is shown in Figure 2. At 3 months and 1 year, the proportion of patients achieving functional independence remained unchanged across the entire population (difference in proportions, 1.1%; 95% CI, −6.9% to 9.2%). An improvement in functional independence was observed in patients with autoimmune encephalitis (difference in proportions, 8.9%; 95% CI, 1.2% to 16.6%), whereas no significant changes were seen in patients with infectious causes (difference in proportions, 1.2%; 95% CI, −6.9% to 9.2%), other causes (difference in proportions, 1.2%; 95% CI, −6.8% to 9.2%), or encephalitis of unknown origin (difference in proportions, −1.9%; 95% CI, −10.0% to 6.2%) (eTable 7 in Supplement 1).

**Figure 3.**
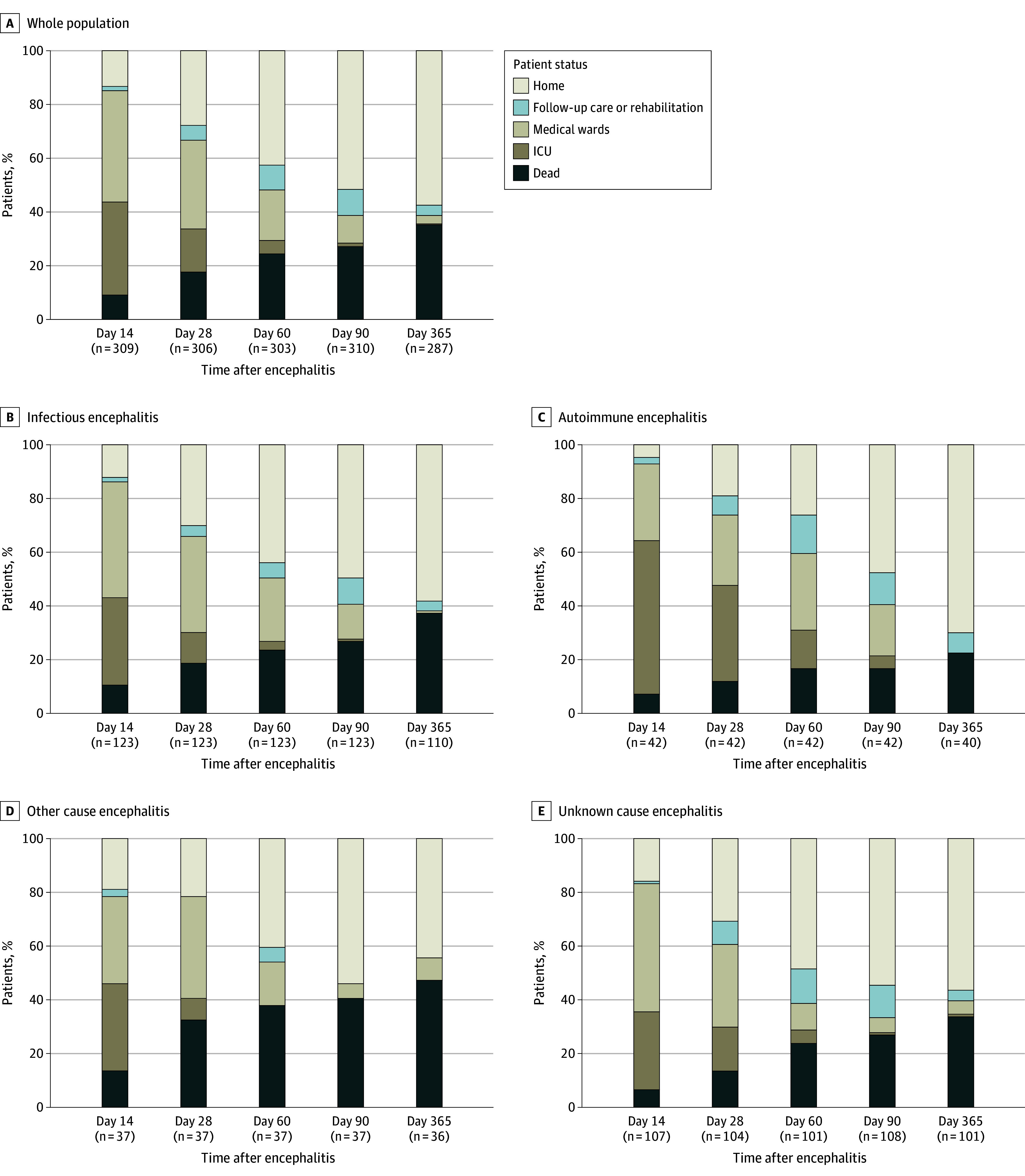
Patient Location up to 1 Year After Severe Encephalitis ICU indicates intensive care unit.

The distribution of places of residence at different time points for the entire cohort and the different causes of encephalitis is displayed in Figure 3. The proportion of patients discharged home at 3 months and 1 year remained unchanged across the entire population (difference in proportions, 5.9%; 95%CI, −2.1% to 13.9%). An improvement in the rate of patients discharged home was observed in patients with infectious encephalitis (difference in proportions, 8.6%; 95% CI, 0.6% to 16.6%), autoimmune encephalitis (difference in proportions, 22.4%; 95% CI, 14.7% to 30.1%), but not with other causes (difference in proportions, −9.7%; 95% CI, −17.7% to −1.7%), or encephalitis of unknown origin (difference in proportions, 2.4%; 95% CI, −5.6% to 10.4%) (eTable 8 in Supplement 1).

## Discussion

The outcomes of patients with encephalitis requiring care in the ICU as examined in the ENCEPHALITICA cohort study reveal several key insights that have important implications for clinical practice. We observed a high incidence of disability or death in this population of patients with severe disease. Specifically, functional independence at 3 months was observed in only 1 in 2 patients, irrespective of the cause of encephalitis. Although we did not observe significant changes in functional recovery rates between 3 months and 1 year in the whole cohort, recovery trajectories varied significantly depending on the underlying cause of encephalitis. Patients diagnosed with autoimmune encephalitis demonstrated more favorable long-term outcomes compared with those with other causes.

Our analyses identified several indicators of outcome in this population of adults with severe all-cause encephalitis. Specifically, older age and immunocompromised status at admission identified patients at very high-risk of poor prognosis at 3 months. Previous studies showed that elderly patients account for a large proportion of adult patients hospitalized with encephalitis, with a high rate of varicella-zoster virus infections, that are known to be associated with a poor prognosis.^26^ In immunocompromised hosts, encephalitis may be caused by atypical pathogens, resulting in delayed diagnosis and appropriate therapies. Moreover, altered immune responses in these patients is associated with atypical presentation, and more severe sequelae.^27^

Empirical treatment with intravenous acyclovir is recommended in any patient with suspected encephalitis.^28^ Of note, previous studies identified that early acyclovir initiation was associated with better outcomes in patients with HSE.^29^ In our study, initiation of empiric acyclovir therapy on the day of ICU admission was shown to be independently associated with higher odds of favorable outcomes in the whole encephalitis population. Acyclovir is a potent antiviral agent that inhibits viral DNA synthesis, effectively reducing viral replication and limiting the extent of brain tissue damage in patients with HSE, and in patients with varicella-zoster virus encephalitis. In other patients, early acyclovir initiation likely reflects an early identification of the encephalitis syndrome, which in turns may translate into faster causal diagnosis and management. Finally, our analyses also identified the non-neurological SOFA score as an independent indicator of the outcome. In contrast, the reason for ICU admission and the severity of neurological presentation, including GCS score and presence of seizure or status epilepticus at admission were not retained in the final multivariable analyses. These results reinforce the impact of non-neurological complications on functional outcomes in this population of severely affected patients, as observed in previous single-center studies.^13,18^

The results from our study highlight substantial heterogeneity among encephalitis causes with respect to 1-year outcomes. Patients with autoimmune encephalitis had the best recovery trajectories, with significant improvement in functional independence between 3 months and 1 year, as compared with other encephalitis causes. These individuals were generally younger, had fewer comorbidities, were more commonly admitted for seizures or status epilepticus, and exhibited lower rates of non-neurological organ failure upon ICU admission. Although they required prolonged mechanical ventilation and extended ICU stays, their 3-month outcomes were comparable, and they showed superior recovery trajectories at the 1-year follow-up compared with other encephalitis causes. These results align with results of previous studies, which reported excellent functional outcome at 24 months in patients withs severe anti-NMDAR encephalitis.^9^ Home discharge rates also improved significantly between 3 months and 1 year in patients with infectious and autoimmune causes of encephalitis, highlighting a potential important role of post-ICU care to reduce persistent disability in these patients.

### Strengths and Limitations

Our study has several strengths, including a large, multicenter setting across 31 sites, which enhances the generalizability of the findings, as well as a prospective data collection. Encephalitis cases were investigated according to international guidelines and adjudicated by an independent committee. We performed rigorous prospective follow-up up to 1 year after the encephalitis diagnosis.

Our study also has limitations. One notable limitation is the lack of centralization for CSF and serum diagnostic studies across the participating centers, which may introduce variability in the encephalitis diagnosis. However, all centers were encouraged to follow guidelines for diagnosis and management of encephalitis, with easy access to microbiological and immunological diagnostic tools. Moreover, all files from included patients were reviewed by an independent adjudication committee. Additionally, the sample size, while adequate for identifying independent outcome indicators at 3 months in the whole population, may limit the ability to detect subtler differences between causes of encephalitis or to generalize findings from less common forms of encephalitis. We acknowledge that extended follow-up could provide valuable insights, particularly in patients without a definitive cause of encephalitis at 1 year. In this cohort, autoimmune causes of encephalitis were relatively uncommon, and the potential contribution of long-term follow-up to the identification of relapses and refinement of encephalitis cause classification, especially in suspected autoimmune cases, remains uncertain. Notably, specific subtypes of autoimmune encephalitis, including leucine-rich glioma-inactivated-11-antibody encephalitis and autoimmune glial fibrillary acidic protein astrocytopathy, were infrequently identified in our cohort of severely affected patients.

## Conclusions

In this cohort study, we observed a poor prognosis at 3 months in one-half of adult patients with severe encephalitis requiring care in the ICU. We observed significant variability in recovery trajectories at 1 year depending on the cause of encephalitis. Specifically, patients with autoimmune encephalitis experienced more favorable outcomes, as compared with other encephalitis groups, suggesting a possible role for targeted long-term support in these cases.

## Notes

Supplement 1. **eTable 1.** Etiological Groups and Definitions **eTable 2.** Participating Centers
eFigure. Flowchart
**eTable 3.** Baseline Characteristics of Excluded Patients **eTable 4.** Secondary Outcomes **eTable 5.** Baseline Characteristics and Their Association With Unfavorable Outcome at Three Months **eTable 6.** Factors Associated With Unfavorable Outcome at Three Months, Univariable and Multivariable Logistic Regression Analyses **eTable 7.** Comparison of Functional Independence Rates at Three Months and One Year **eTable 8.** Comparison of Home Discharge Rates at Three Months and One Year

Supplement 2.
Nonauthor Collaborators

Supplement 3.
Data Sharing Statement
