# Supplementary material for: Outcomes of Critically Ill Adult Patients With Acute Encephalitis
Source: JAMA Netw Open. 2025 Sep 18;8(9):e2532478. doi: 10.1001/jamanetworkopen.2025.32478 (PMC12447255; doi:10.1001/jamanetworkopen.2025.32478)
Supplement: Supplement 1. — eTable 1. Etiological Groups and Definitions eTable 2. Participating Centers eFigure. Flowchart eTable 3. Baseline Characteristics of Excluded Patients eTable 4. Secondary Outcomes eTable 5. Baseline Characteristics and Their Association With Unfavorable Outcome at Three Months eTable 6. Factors Associated With Unfavorable Outcome at Three Months, Univariable and Multivariable Logistic Regression Analyses eTable 7. Comparison of Functional Independence Rates at Three Months and One Year eTable 8. Comparison of Home Discharge Rates at Three Months and One Year [file jamanetwopen-e2532478-s001.pdf]

Supplemental Online Content

Sonneville R, Couffignal C, Souweine B, et al. Outcomes of critically ill adult patients with acute encephalitis. *JAMA Netw. Open*. 2025;8(9):e2532478.
doi:10.1001/jamanetworkopen.2025.32478

eTable 1. Etiological Groups and Definitions
eTable 2. Participating Centers
eFigure. Flowchart
eTable 3. Baseline Characteristics of Excluded Patients
eTable 4. Secondary Outcomes
eTable 5. Baseline Characteristics and Their Association With Unfavorable Outcome at Three Months
eTable 6. Factors Associated With Unfavorable Outcome at Three Months, Univariable and Multivariable Logistic Regression Analyses
eTable 7. Comparison of Functional Independence Rates at Three Months and One Year
eTable 8. Comparison of Home Discharge Rates at Three Months and One Year

This supplemental material has been provided by the authors to give readers additional information about their work.

© 2025 Sonneville R et al. *JAMA Network Open*.

eTable 1. Etiological groups and definitions

Etiological groups	Definitions
Infectious Encephalitis	Detection of an appropriate pathogen by CSF PCR, metagenomics, mycobacterial, or fungal culture.
Autoimmune Encephalitis	Detection of autoantibodies in serum or CSF, or evidence of acute demyelination on brain MRI<br>OR<br>Criteria for autoimmune seronegative encephalitis.<br>Reasonable exclusion of common infectious causes.
Other causes	Neoplastic: Evidence of neoplastic cells in CSF.<br>Metabolic/toxic: severe metabolic disturbances +/- recent exposure to drug(s) with known neurotoxicity.<br>Reasonable exclusion of common infectious causes.
Encephalitis of Unknown Origin	No cause identified.<br>Reasonable exclusion of common infectious causes.

© 2025 Sonneville R et al. *JAMA Network Open*.

eTable 2. Participating centers

Centers	Number of patients
Bichat - Paris - Réanimation médicale	41
Clermont-Ferrand - Réanimation médicale	33
La Tronche - Réanimation médicale	21
Henri Mondor - Créteil - Réanimation médicale	19
CH André Mignot - Le Chesnay - Réanimation médico-chirurgicale	17
Nantes - CHU hôtel dieu - Réanimation médicale	17
Pitié Salpêtrière - Paris - Réanimation médicale	16
Pitié-Salpêtrière - Paris - Réanimation médicale à orientation neurologique	15
La cavale blanche - Brest - Réanimation médicale	13
Cochin - Paris - Réanimation médicale	10
Metz -Thionville - Réanimation médico-chirurgicale	10
Nancy - central - Réanimation médicale	9
Tenon - Paris - Réanimation médico-chirurgicale	9
Poitiers - Réanimation médicale	9
Marseille - APHM Sainte-Marguerite - Réanimation médicale	8
Rennes - CHU Pontchaillou – Réanimation médicale	8
Angers - Réanimation médicale	7
Argenteuil - CH victor dupouy – Réanimation médico-chirurgicale	7
Besançon - CHU Jean Minjoz - Réanimation médicale	5
Bron - hôpital Pierre Wertheimer – Réanimation médico-chirurgicale	5
Lyon - hôpital Edouard Herriot - Réanimation médicale	5
Pitié Salpêtrière - Paris - institut de cardiologie - Réanimation médicale #2	5
Saint Antoine - Paris - Réanimation médicale	4
Avicenne - Bobigny - Réanimation médico-chirurgicale	3
Lille - CHRU Roger Salengro - Réanimation	3
Suresnes - hôpital Foch - Réanimation médico-chirurgicale	3
Boulogne - Ambroise Paré - Réanimation médico-chirurgicale	2
Saint-Joseph - Paris - Réanimation médico-chirurgicale	2
Saint Louis - Paris - Réanimation médicale	2
Louis Mourier - Réanimation médico-chirurgicale	1
Roanne - CH de Roanne – Réanimation médicale	1

© 2025 Sonneville R et al. *JAMA Network Open*.

eFigure. Flowchart

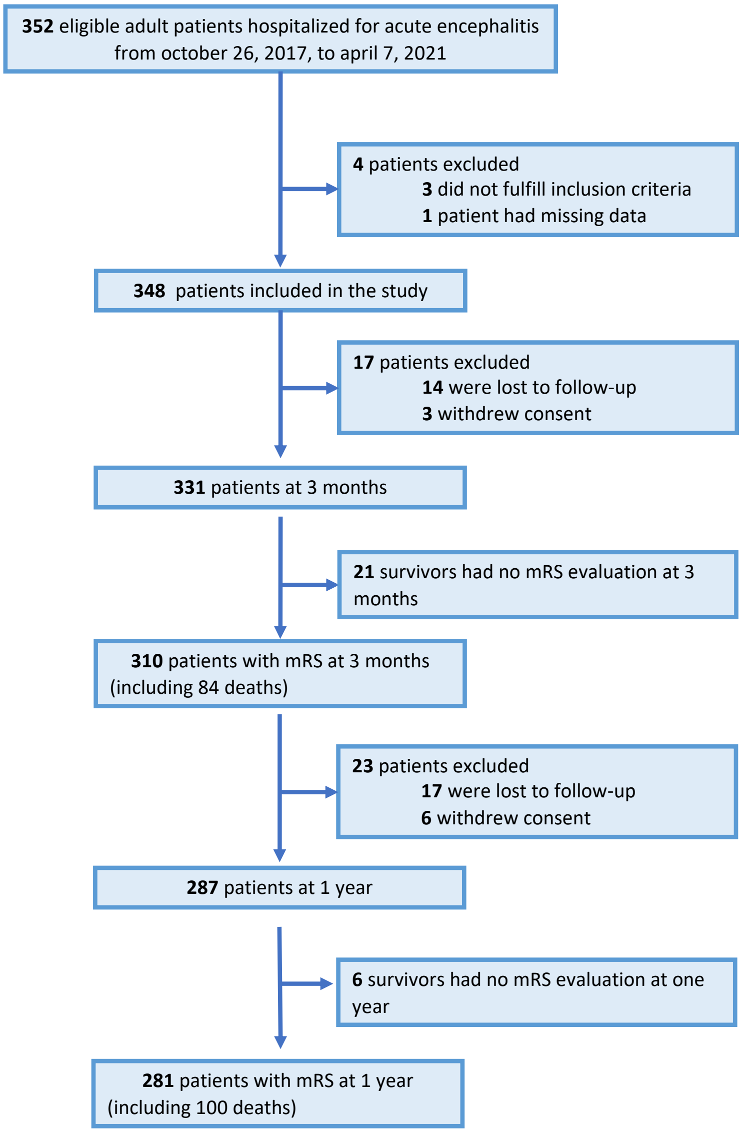

© 2025 Sonneville R et al. *JAMA Network Open*.

eTable 3. Baseline characteristics of excluded patients (n=38)

	Included patients *(n=310)*	Excluded patients *(n=38)*
**Age, years**	60 [43—72]	63 [52—72]
**Gender, male**	177/310 (57.1)	22/38 (57.9)
**Charlson comorbidity index**	3 [1—6]	3 [2—5]
**Immunodepression***	74/310 (23.9)	6/38 (15.8)
**Reason for ICU admission**		
Altered mental status	178/309 (57.6)	21/38 (55.3)
Seizures / status epilepticus	69/309 (22.3)	9/38 (23.7)
Sepsis / respiratory failure	10/309 (3.2)	1/38 (2.6)
Other	38/309 (12.3)	3/38 (7.9)
**Glasgow coma scale**		
Score	10 [7—12]	9 [6—11]
Eye component	3 [1—4]	3 [1—3]
Verbal component	2 [1—3]	2 [1—3]
Motor component	5 [4—6]	4 [3—6]
**Temperature**		
Degrees, °C	37.8 [36.9—38.7]	37.4 [36.3—38.3]
≥38.0°C, indicating fever	239/310 (77.1)	29/38 (76.3)
**Seizures**	142/310 (45.8)	16/38 (42.1)
**Focal neurologic findings**	163/310 (52.6)	14/38 (36.8)
**SAPS 2**	42 [29—57]	50 [34—59]
**Non-neurologic SOFA score**	3 [1—5]	3 [1—6]
**CSF**		
Time from inclusion to LP, days	0 [0—1]	0 [-1—0]
CSF leucocytes ≥5/mm^3	221/277 (79.8)	26/35 (74.3)
Leucocytes, cells/mm^3	18 [6—93]	10 [3.5—80.5]
Lymphocytes, %	74 [17—92]	64 [11—80]
Protein level, g/L	0.7 [0.5—1.3]	0.6 [0.4—1]
Glucose, mmol/L	3.9 [3.3—5]	4.3 [3.8—4.8]
**Brain imaging**		
CT-scan performed	266/309 (86.1)	31/38 (81.6)
Time from inclusion to CT-scan, days	0 [-1—0]	0 [0—0]
Abnormal CT-scan	85/263 (32.3)	6/31 (19.4)
MRI performed	223/309 (72.2)	29/38 (76.3)
Time from inclusion to MRI, days	1 [0—2]	1 [-1—2]
Abnormal MRI	162/223 (72.6)	19/29 (65.5)
**EEG**		
EEG performed	273/310 (88.1)	35/38 (92.1)
Time from inclusion to EEG, days	1 [0—1]	0 [0—1]
Abnormal EEG	142/310 (45.8)	11/38 (28.9)

Data are median [IQR] or n (%). ICU intensive care unit; CSF cerebrospinal fluid; SAPS, Simplified Acute Physiology Score; SOFA, Sequential Organ Failure Assessment. mRS modified Rankin scale. MRI magnetic resonance imaging; EEG Electroencephalography.

© 2025 Sonneville R et al. *JAMA Network Open*.

eTable 4. Secondary outcomes

	Total *(n=310)*	Infectious *(n=123)*	Autoimmune *(n=42)*	*Other (n=37)*	Unknown (n=108)	P value
**ICU stay**						
Received acyclovir	223/276 (80.8)	88/116 (75.9)	31/32 (96.9)	30/34 (88.2)	74/94 (78.7)	0.02
Received steroids	71/309 (23)	18/123 (14.6)	29/42 (69)	7/37 (18.9)	17/107 (15.9)	<0.001
Received intravenous immunoglobulins	31/308 (10.1)	6/123 (4.9)	19/41 (46.3)	2/37 (5.4)	4/107 (3.7)	<0.001
Received plasma exchange	14/308 (4.5)	2/122 (1.6)	9/42 (21.4)	1/37 (2.7)	2/107 (1.9)	<0.001
Received invasive mechanical ventilation	227/309 (73.5)	87/123 (70.7)	32/42 (76.2)	28/37 (75.7)	80/107 (74.8)	0.87
Duration of invasive ventilation, days	6 [3—17]	6 [3—14]	18 [8—32]	5 [3—11]	6 [3—15]	<0.001
Duration of ICU stay, days	9 [5—19]	10 [5—18]	19 [7—43]	9 [6—15]	8 [5—16]	0.002
Decisions to withhold or withdraw care	46/308 (14.9)	18/122 (14.8)	7/42 (16.7)	7/37 (18.9)	14/107 (13.1)	0.79
**ICU discharge**						
Mortality	54/310 (17.4)	21/123 (17.1)	7/42 (16.7)	9/37 (24.3)	17/108 (15.7)	0.68
Medical wards	238/256 (93)	100/102 (98)	30/35 (85.7)	25/28 (89.3)	83/91 (91.2)	0.02
Follow-up care or rehabilitation	15/256 (5.9)	2/102 (2)	5/35 (14.3)	1/28 (3.6)	7/91 (7.7)	0.04
Home	3/256 (1.2)	0/102 (0)	0/35 (0)	2/28 (7.1)	1/91 (1.1)	0.03
**Three-month outcomes**						
Functional independence (mRS 0-2)	161/310 (51.9)	64/123 (52)	25/42 (59.5)	16/37 (43.2)	56/108 (51.9)	0.56
At home	160/310 (51.6)	61/123 (49.6)	20/42 (47.6)	20/37 (54.1)	59/108 (54.6)	0.81
**One-year outcomes**						
Functional independence (mRS 0-2)	149/281 (53)	58/109 (53.2)	26/38 (68.4)	16/36 (44.4)	49/98 (50)	0.17
At home	165/287 (57.5)	64/110 (58.2)	28/40 (70)	16/36 (44.4)	57/101 (56.4)	0.20

Data are median [IQR] or n (%). mRS modified Rankin Scale; ICU, Intensive Care Unit; SAPS, Simplified Acute Physiology Score; SOFA, Sequential Organ Failure Assessment. mRS modified Rankin scale.

eTable 5. Baseline characteristics and their association with unfavorable outcome at three months

	**Total** *(n=310)*	**mRS 0-2** *(n=161)*	**mRS 3-6** *(n=149)*	**P value**
**Age, years**	60 [43—72]	53 [37—66]	68 [55—77]	<0.001
**Male gender**	177 (57)	92 (57)	85 (57)	>0.99
**Immunodepression***	74 (24)	25 (16)	49 (33)	<0.001
**Reason for ICU admission**				0.004
Altered mental status	178 (58)	91 (57)	87 (59)	.
Seizures / status epilepticus	69 (22)	44 (27)	25 (17)	.
Sepsis / respiratory failure	24 (8)	5 (3)	19 (13)	.
Other	38 (12)	21 (13)	17 (12)	.
**Glasgow coma scale**				
Score	10 [7—12]	10 [7—12]	10 [6—12]	0.94
Eye component	3 [1—4]	3 [1—4]	3 [1—4]	0.87
Verbal component	2 [1—3]	2 [1—3]	2 [1—3]	0.84
Motor component	5 [4—6]	5 [4—6]	5 [4—5]	0.38
**Temperature**				
Degrees, °C	37.8 [36.9—38.7]	37.8 [37—38.9]	37.6 [36.8—8.6]	0.23
≥38.0°C, indicating fever	239 (77)	130 (81)	109 (73)	0.11
**Seizures**	142 (46)	75 (47)	67 (45)	0.77
**Focal neurologic findings**	163 (53)	82 (51)	81 (54)	0.55
**SAPS 2, points**	42 [29—57]	37 [25—51]	50 [35—60]	<0.001
**Non-neurologic SOFA score**	3 [1—5]	2 [1—4]	3 [2—6]	<0.001
Respiratory component >2[b]	47 (16)	18 (12)	29 (20)	0.06
Cardiovascular component >2[c]	57 (19)	21 (14)	36 (25)	0.01
Renal component >2[d]	38 (15)	16 (10)	22 (15)	0.22
Liver component >2[e]	0 (0)	0 (0)	0 (0)	.
Coagulation component >2[f]	8 (3)	4 (3)	4 (3)	0.90
**CSF**				
Time from inclusion to LP, days	0 [0—2]	0 [0—2]	0 [0—3]	0.02
CSF leucocytes ≥5/mm^3	249 (80)	130 (81)	119 (80)	0.85
Leucocytes, cells/mm^3	18 [6—93]	20 [6—93]	18 [6—92]	0.82
Lymphocytes, %	74 [17—92]	82 [11—94]	69 [25—90]	0.39
Protein level, g/L	0.7 [0.5—1.3]	0.6 [0.4—1.1]	0.7 [0.5—1.5]	0.02
Glucose, mmol/L	3.9 [3.3—5.0]	4.2 [3.4—5.2]	3.9 [3.0—5.0]	0.08
**Brain imaging**				
CT-scan performed	266/309 (86)	138/161 (86)	128/148 (87)	0.85
Time from inclusion to CT-scan, days	0 [-1—0]	0 [-1—0]	0 [0—0]	0.03
Abnormal CT-scan	85/263 (32)	35/137 (26)	50/126 (40)	0.01
MRI performed	223/309 (72)	121/161 (75)	102/148 (69)	0.22
Time from inclusion to MRI, days	1 [0—2]	1 [0—2]	1 [0—3]	0.49

© 2025 Sonneville R et al. *JAMA Network Open*.

Abnormal MRI	162/223 (73)	77/121 (64)	85/102 (83)	0.001
**EEG**				
EEG performed	273/308 (89)	140/160 (88)	133/148 (90)	0.51
Time from inclusion to EEG, days	1 [0—1]	1 [0—1]	1 [0—2]	0.07
Abnormal EEG	142/273 (46)	71/140 (44)	71/133 (48)	0.53

Data are median [IQR] or n (%). mRS modified Rankin Scale; ICU, Intensive Care Unit; SAPS, Simplified Acute Physiology Score; SOFA, Sequential Organ Failure Assessment.

*Immunodepression includes patients living with HIV (n=25), solid organ transplantation (n=17), cancer or hematology disease (n=25), and/or steroids/immunosuppressants (n=27).

[a]Altered mental status is defined by a GCS score ≤ 13.

[b]A respiratory SOFA score >2 indicates a PaO_2/FiO_2 ratio <200 with respiratory support.

[c]A cardiovascular SOFA score >2 indicates the use of norepinephrine or epinephrine (any dose).

[d]A renal SOFA score >2 indicates a serum creatinine >300 µmol/L

[e]A liver SOFA score >2 indicates a bilirubin concentration >100 µmol/L

[f]A coagulation SOFA score >2 indicates a platelet count <50 platelets x 10^3/µL

© 2025 Sonneville R et al. *JAMA Network Open*.

eTable 6. Factors associated with unfavorable outcome at three months, univariable and multivariable logistic regression analyses

				Univariable analysis		Full Model		Multivariable analysis	
**Risk factors**	**Total N = 310**	**mRS [0-2] N = 161**	**mRS [3-6] N = 149**	**OR**	**p-value**	**aOR**	**p-value**	**aOR**	**p-value**
**Age, OR by 5-year increment**	57.7 (18.3)	51.1 (17.8)	68 [55:77]	1.26 [1.17-1.36]	6.00×10^{-10}	1.28 [1.16-1.41]	1.00×10^{-6}	1.28 [1.18-1.40]	<0.001
**Immunocompromised**	74/310 (23.9)	25/161 (15.5)	49/149 (32.9)	2.67 [1.56-4.66]	0.0004	3.12 [1.57-6.40]	0.001	3.02 [1.63-5.77]	<0.001
**Score on the GCS, OR per one-point increment**	9 (3.2)	9 (3.3)	9 (3.2)	1.00 [0.94-1.07]	0.94	1.04 [0.94-1.16]	0.42		
Focal signs	163/310 (52.6)	82/161 (50.9)	81/149 (54.4)	1.15 [0.73-1.80]	0.55	0.95 [0.53-1.69]	0.86		
**Seizures**	142/310 (45.8)	75/161 (46.6)	67/149 (45)	0.94 [0.60-1.47]	0.78	0.82 [0.45-1.48]	0.51		
Fever	239/310 (77.1)	130/161 (80.7)	109/149 (73.2)	0.65 [0.38-1.11]	0.11	0.80 [0.39-1.61]	0.53		
**CSF pleocytosis**	249/310 (80.3)	130/161 (80.7)	119/149 (79.9)	0.95 [0.54-1.66]	0.85	0.92 [0.44-1.96]	0.84		
**Abnormal brain imaging**	131/310 (42.3)	61/161 (37.9)	70/149 (47)	1.45 [0.92-2.29]	0.11	1.67 [0.91-3.07]	0.098		
**Abnormal EEG findings**	142/310 (45.8)	71/161 (44.1)	71/149 (47.7)	1.15 [0.74-1.81]	0.53	1.11 [0.62-1.99]	0.73		
**SAPS2, OR per one-point increment**	44.7 (19.2)	39.6 (17.4)	50.2 (19.6)	1.03 [1.02-1.05]	7.00×10^{-6}	1.01 [0.99-1.03]	0.51		
**Non-neurological SOFA score, OR per one-point increment**	3.5 (3)	2.9 (2.8)	4.1 (3.1)	1.15 [1.06-1.25]	0.0007	1.12 [1.01-1.26]	0.037	1.10 [1.01-1.20]	0.04
**IV acyclovir within 24hours**	204/310 (65.8)	117/161 (72.7)	87/149 (58.4)	0.53 [0.33-0.85]	0.008	0.38 [0.20-0.72]	0.003	0.36 [0.20-0.63]	<0.001
**IV3rd generation cephalosporin within 24 hours**	109/310 (35.2)	63/161 (39.1)	46/149 (30.9)	0.69 [0.43-1.11]	0.13	0.89 [0.48-1.67]	0.72		
**Aetiologies**									
Infectious	123/310 (39.7)	64/161 (39.8)	59/149 (39.6)	1.00	0.55	1.00	0.51		
Other causes	37/310 (11.9)	16/161 (9.9)	21/149 (14.1)	1.42 [0.68-3.02]		1.15 [0.43-3.13]			
Autoimmune	42/310 (13.5)	25/161 (15.5)	17/149 (11.4)	0.74 [0.36-1.49]		1.98 [0.78-5.10]			
Unknown	108/310 (34.8)	56/161 (34.8)	52/149 (34.9)	1.01 [0.60-1.69]		1.01 [0.51-2.00]			

© 2025 Sonneville R et al. *JAMA Network Open*.

eTable 7. Comparison of functional independence rates at three months and one year

Category	Functional independence at three months	Functional independence at one year	Difference in proportion (95%CI)	p-value
All patients (n=310)	51.9 %	53.0%	1.1% (-6.9% to 9.2%)	0.79
Infectious (n=123)	52.0 %	53.2%	1.2% (-6.9% to 9.2%)	0.77
Autoimmune (n=42)	59.5 %	68.4%	8.9% (1.2% to 16.6%)	0.01
Other causes (n=37)	43.2 %	44.4%	1.2% (-6.8% to 9.2%)	0.77
Unknown origin (n=108)	51.9 %	50.0%	-1.9% (-10.0% to 6.2%)	0.64

© 2025 Sonneville R et al. *JAMA Network Open*.

eTable 8. Comparison of home discharge rates at three months and one year

Category	Home discharge at three months	Home discharge at one year	Difference in proportion (95%CI	p-value
All patients (n=310)	51.6 %	57.5%	5.9% (-2.1% to 13.9%)	0.15
Infectious causes (n=123)	49.6 %	58.2%	8.6% (0.6% to 16.6%)	0.04
Autoimmune causes (n=42)	47.6 %	70.0%	22.4% (14.7% to 30.1%)	<0.001
Other causes (n=37)	54.1 %	44.4%	-9.7% (-17.7% to -1.7%)	0.02
Unknown origin (n=108)	54.6 %	56.4%	2.4% (-5.6% to 10.4%)	0.56

© 2025 Sonneville R et al. *JAMA Network Open*.
