# Supplementary material for: Outcomes of Critically Ill Adult Patients With Acute Encephalitis
Source: JAMA Netw Open. 2025 Sep 18;8(9):e2532478. doi: 10.1001/jamanetworkopen.2025.32478 (PMC12447255; doi:10.1001/jamanetworkopen.2025.32478)
Supplement: Supplement 2. — Nonauthor Collaborators [file jamanetwopen-e2532478-s002.pdf]

Supplemental Online Content: Nonauthor Collaborators
*First name, last name, and suffix (if applicable) are required and will appear in PubMed.

| *Group Name(s): ENCEPHALITICA Investigator Study Group | | | | | | | |
|---|---|---|---|---|---|---|---|
| ***First Name and Middle Initial(s)** | ***Last Name** | ***Suffix (eg, Jr, III)** | Academic Degrees | Institution | Location (city, state/province, country) | Role or Contribution, eg, chair, principal investigator | Group (if more than 1 Group listed in the byline) and/or Subgroup (eg, Steering Committee) |
| Moustafa | ABDEL-NABEY | | | | | | |
| Melanie | ADDA | | | | | | |
| Hamou Zakaria | AIT | | | | | | |
| Virginie | ANZANO | | | | | | |
| Romain | ARRESTIER | | | | | | |
| Pierre | ASFAR | | | | | | |
| Claire | BACHELIER | | | | | | |
| François | BAGATE | | | | | | |
| Elodie | BARON | | | | | | |
| Thomas | BAUDRY | | | | | | |
| François | BELONCLE | | | | | | |
| Morgan | BENAIS | | | | | | |
| Ines | BENDIB | | | | | | |
| Brice | BENELLI | | | | | | |
| Sarah | BENGHANEM | | | | | | |
| Helene | BERINGUER | | | | | | |
| Enora | BERTI | | | | | | |
| Astrid | BERTIER | | | | | | |
| Morage | BERTRIX | | | | | | |
| Sebastien | BESSET | | | | | | |
| Alexandra | BEURTON | | | | | | |
| Naike | BIGE | | | | | | |
| Clarisse | BLAYAU | | | | | | |
| Florence | BOISSIER | | | | | | |
| Pierre-Edouard | BOLLAERT | | | | | | |
| Marjolaine | BOREL | | | | | | |
| Lila | BOUADMA | | | | | | |
| Athenais | BOUCLY | | | | | | |
| Simon | BOURCIER | | | | | | |

Supplemental Online Content: Nonauthor Collaborators
*First name, last name, and suffix (if applicable) are required and will appear in PubMed.

| ***First Name and Middle Initial(s)** | ***Last Name** | ***Suffix (eg, Jr, III)** | Academic Degrees | Institution | Location (city, state/province, country) | Role or Contribution, eg, chair, principal investigator | Group (if more than 1 Group listed in the byline) and/or Subgroup (eg, Steering Committee) |
|---|---|---|---|---|---|---|---|
| Radhia | BOUZGARROU | | | | | | |
| Cedric | BRUEL | | | | | | |
| Côme | BUREAU | | | | | | |
| Cyril | CADOZ | | | | | | |
| Laure | CALVET | | | | | | |
| Emmanuel | CANET | | | | | | |
| Albert | CAO | | | | | | |
| Alain | CARIOU | | | | | | |
| Romain | CARRILLON | | | | | | |
| Pedro | CAVALEIRO | | | | | | |
| Julien | CHARPENTIER | | | | | | |
| Delphine | CHATELLIER | | | | | | |
| Vibol | CHHOR | | | | | | |
| Jean-Daniel | CHICHE | | | | | | |
| Sebastien | CLERC | | | | | | |
| Benjamin | COIFFARD | | | | | | |
| Remi | COUDROY | | | | | | |
| Pierre | COUHAULT | | | | | | |
| Elisabeth | COUPEZ | | | | | | |
| Guilhem | COURTE | | | | | | |
| Aurélie | CRAVOISY-POPOVIC | | | | | | |
| Thibault | CREUTIN | | | | | | |
| Laura | CROSBY | | | | | | |
| Daniel | DA SILVA | | | | | | |
| Florence | DAVIET | | | | | | |
| Etienne | DE MONTMOLLIN | | | | | | |
| Maxens | DECAVELE | | | | | | |
| Thecle | DEGROOTE | | | | | | |
| Robin | DELERIS | | | | | | |
| Sophie | DEMERET | | | | | | |
| Suela | DEMIRI | | | | | | |

Supplemental Online Content: Nonauthor Collaborators
*First name, last name, and suffix (if applicable) are required and will appear in PubMed.

| ***First Name and Middle Initial(s)** | ***Last Name** | ***Suffix (eg, Jr, III)** | Academic Degrees | Institution | Location (city, state/province, country) | Role or Contribution, eg, chair, principal investigator | Group (if more than 1 Group listed in the byline) and/or Subgroup (eg, Steering Committee) |
|---|---|---|---|---|---|---|---|
| Julien | DEMISELLE | | | | | | |
| Martin | DRES | | | | | | |
| Louis-Marie | DUMONT | | | | | | |
| Julien | DUPEYRAT | | | | | | |
| Pierre | DUPLAND | | | | | | |
| Claire | DUPUIS | | | | | | |
| Ahmed | EL KALIOUBIE | | | | | | |
| Morgane | FAURE | | | | | | |
| Laura | FEDERICI | | | | | | |
| Alexis | FERRE | | | | | | |
| Jean-Marie | FOREL | | | | | | |
| Guillaume | FRANCHINEAU | | | | | | |
| Jean-Pierre | FRAT | | | | | | |
| Santiago | FREITA-RAMOS | | | | | | |
| Rostane | GACI | | | | | | |
| Arnaud | GACOUIN | | | | | | |
| Aude | GARIN | | | | | | |
| Charlotte | GARRET | | | | | | |
| Ariane | GAVAUD | | | | | | |
| Segolene | GENDREAU | | | | | | |
| Etienne | GHRENASSIA | | | | | | |
| Sebastien | GIBOT | | | | | | |
| Armelle | GILARD | | | | | | |
| Tiphaine | GIRARD | | | | | | |
| Florent | GOBERT | | | | | | |
| Kevin | GRAPIN | | | | | | |
| Christophe | GUERVILLY | | | | | | |
| Marion | HALLARD | | | | | | |
| Geoffroy | HARIRI | | | | | | |
| Otto | HARTMAN | | | | | | |
| Anne-Fleur | HAUDEBOURG | | | | | | |

Supplemental Online Content: Nonauthor Collaborators
*First name, last name, and suffix (if applicable) are required and will appear in PubMed.

| *First Name and Middle Initial(s) | *Last Name | *Suffix (eg, Jr, III) | Academic Degrees | Institution | Location (city, state/province, country) | Role or Contribution, eg, chair, principal investigator | Group (if more than 1 Group listed in the byline) and/or Subgroup (eg, Steering Committee) |
|---|---|---|---|---|---|---|---|
| Luc | HAUDEBOURG | | | | | | |
| Jeremy | HEBRAUD | | | | | | |
| Jérôme | HONNORAT | | | | | | |
| Pierre | JAQUET | | | | | | |
| Paul | JAUBERT | | | | | | |
| Florent | JOLY | | | | | | |
| Matthieu | JOZWIAK | | | | | | |
| Hélène | JULIEN | | | | | | |
| Francis | KINDA | | | | | | |
| Pascale | LABEDADE | | | | | | |
| Pascale | LABROCA | | | | | | |
| Guillaume | LACAVE | | | | | | |
| Driss | LAGHLAM | | | | | | |
| Pauline | LAMOUCHE-WILQUIN | | | | | | |
| Jean-Baptiste | LASCARROU | | | | | | |
| Virginie | LAURENT | | | | | | |
| Guillaume | LAURICHESSE | | | | | | |
| Guennec Loïc | LE | | | | | | |
| Marec Julien | LE | | | | | | |
| Marie | LECRONIER | | | | | | |
| Lucie | LEFEVRE | | | | | | |
| Camille | LEGOUY | | | | | | |
| Stephane | LEGRIEL | | | | | | |
| Jérémie | LEMARIE | | | | | | |
| Marie | LEMERLE | | | | | | |
| Jean-Mathias | LITEAUDON | | | | | | |
| Julien | LOPINTO | | | | | | |
| Charles-Edouard | LUYT | | | | | | |
| Adel | MAAMAR | | | | | | |
| Assadi | MAKSUD | | | | | | |
| Damien | MARIE | | | | | | |

Supplemental Online Content: Nonauthor Collaborators
*First name, last name, and suffix (if applicable) are required and will appear in PubMed.

| *First Name and Middle Initial(s) | *Last Name | *Suffix (eg, Jr, III) | Academic Degrees | Institution | Location (city, state/province, country) | Role or Contribution, eg, chair, principal investigator | Group (if more than 1 Group listed in the byline) and/or Subgroup (eg, Steering Committee) |
|---|---|---|---|---|---|---|---|
| Eric | MARIOTTE | | | | | | |
| Remy | MARNAI | | | | | | |
| Olivier | MARTIN | | | | | | |
| Paul | MASI | | | | | | |
| Rafael | MATHIEU | | | | | | |
| Eric | MAURY | | | | | | |
| Julien | MAYAUX | | | | | | |
| Sandie | MAZERAND | | | | | | |
| Alain | MERCAT | | | | | | |
| Sybille | MERCERON | | | | | | |
| Arnaud-Félix | MIAILHE | | | | | | |
| Gael | MICHAUD | | | | | | |
| Hélène | MIGUERES | | | | | | |
| Jean-Paul | MIRA | | | | | | |
| Grégoire | MONSEAU | | | | | | |
| Elise | MORAWIEC | | | | | | |
| Jean | MORIN | | | | | | |
| Satar | MORTAZA | | | | | | |
| Lionel | NACE | | | | | | |
| Safaa | NEMLAGHI | | | | | | |
| Mathilde | NEUVILLE | | | | | | |
| Bao-Long | NGUYEN | | | | | | |
| Lee | NGUYEN | | | | | | |
| Pierre-Yves | OLIVIER | | | | | | |
| Gregoire | OTTAVY | | | | | | |
| Laurent | PAPAZIAN | | | | | | |
| Edwidge | PEJU | | | | | | |
| Frederic | PENE | | | | | | |
| François | PERIER | | | | | | |
| François | PHILIPPART | | | | | | |
| Marc | PIERROT | | | | | | |

Supplemental Online Content: Nonauthor Collaborators
*First name, last name, and suffix (if applicable) are required and will appear in PubMed.

| ***First Name and Middle Initial(s)** | ***Last Name** | ***Suffix (eg, Jr, III)** | Academic Degrees | Institution | Location (city, state/province, country) | Role or Contribution, eg, chair, principal investigator | Group (if more than 1 Group listed in the byline) and/or Subgroup (eg, Steering Committee) |
|---|---|---|---|---|---|---|---|
| Olivier | POULY | | | | | | |
| Gwenael | PRAT | | | | | | |
| Simona | PRESENTE | | | | | | |
| Isabelle | PRIOUR | | | | | | |
| Thomas | RAMBAUD | | | | | | |
| Keyvan | RAZAZI | | | | | | |
| Jean | REIGNIER | | | | | | |
| Anne | RENAULT | | | | | | |
| Thomas | RITZENTHALER | | | | | | |
| Alexandre | ROBERT | | | | | | |
| Benjamin | ROHAUT | | | | | | |
| Guillaume | SAVARY | | | | | | |
| Faiza | SAYAGH | | | | | | |
| Carole | SCHWEBEL | | | | | | |
| Amélie | SEGUIN | | | | | | |
| Stein | SILVA | | | | | | |
| Fabrice | SINNAH | | | | | | |
| Vincent | SOUDAY | | | | | | |
| Françoise | THOUY | | | | | | |
| Jean-Marie | TONNELIER | | | | | | |
| Aurelia | TOUSSAINT | | | | | | |
| Marc | TRAN | | | | | | |
| Samuel | TUFFET | | | | | | |
| Fabrice | UHEL | | | | | | |
| Thomas | URBINA | | | | | | |
| Anne | VEINSTEIN | | | | | | |
| Charles | VERNEY | | | | | | |
| Antoine | VIEILLARD-BARON | | | | | | |
| Clara | VIGNERON | | | | | | |
| Helene | VINOUR | | | | | | |
| Sara | VIROLLE | | | | | | |

Supplemental Online Content: Nonauthor Collaborators
*First name, last name, and suffix (if applicable) are required and will appear in PubMed.

| ***First Name and Middle Initial(s)** | ***Last Name** | ***Suffix (eg, Jr, III)** | Academic Degrees | Institution | Location (city, state/province, country) | Role or Contribution, eg, chair, principal investigator | Group (if more than 1 Group listed in the byline) and/or Subgroup (eg, Steering Committee) |
|---|---|---|---|---|---|---|---|
| Lev | VOLKOV | | | | | | |
| Constance | VUILLARD | | | | | | |
| Nicolas | WEISS | | | | | | |
| Paul Henri | WICKY | | | | | | |
| Lara | ZAFRANI | | | | | | |
| Olivier | ZAMBON | | | | | | |
| Noemie | ZUCMAN | | | | | | |
