# Supplementary material for: Outcomes of Critically Ill Adult Patients With Acute Encephalitis
Source: JAMA Netw Open. 2025 Sep 18;8(9):e2532478. doi: 10.1001/jamanetworkopen.2025.32478 (PMC12447255; doi:10.1001/jamanetworkopen.2025.32478)
Supplement: Supplement 3. — Data Sharing Statement [file jamanetwopen-e2532478-s003.pdf]

Data Sharing Statement

Sonneville. Outcomes of Critically Ill Adult Patients With Acute Encephalitis. *JAMA Netw Open*. Published September 18, 2025. doi:10.1001/jamanetworkopen.2025.32478

Data
Data available: Yes
Data types: Deidentified participant data, Data dictionary
How to access data: Data will be made available upon reasonable request to the corresponding author and contingent upon approval by the institutional review board and a signed data use agreement. Deidentified participant data and a data dictionary will be shared for academic, noncommercial purposes.
When available: With publication

Supporting Documents
Document types: None

Additional Information
Who can access the data: Data will be made available to researchers whose proposed use of the data has been approved.
Types of analyses: Data will be made available for or ancillary studies and/or meta-analyses.
Mechanisms of data availability: Data will be made available after approval of a proposal and after a signed data access agreement has been completed.
Any additional restrictions: none.
